# COVID-19 presenting as fulminant hepatic failure: A case report

**DOI:** 10.1097/MD.0000000000022818

**Published:** 2020-10-23

**Authors:** Stephanie Melquist, Kayla Estepp, Yauheni Aleksandrovich, Angela Lee, Andrea Beiseker, Farid Saei Hamedani, John Bassett

**Affiliations:** a University of North Dakota School of Medicine and Health Sciences, Department of Internal Medicine; b Sanford Health, Department of Internal Medicine, Gastroenterology; c Sanford Health, Department of Pathology, ND.

**Keywords:** COVID-19, SARS-CoV-2, fulminant hepatic failure, hepatitis, lupus

## Abstract

Introduction: Severe acute respiratory syndrome corona virus 2 (SARS-CoV-2) responsible for the COVID-19 pandemic has spread from Wuhan, China in December, 2019 to 216 countries and territories as of September 10, 2020 with 27.74 million cases and 899,911 confirmed deaths. The spectrum of disease is most commonly seen as a viral pneumonia with high grade fevers, shortness of breath, dry cough, and chest pain with radiologic evidence of bilateral, interstitial, ground glass opacities, and peripheral lung consolidation. Liver chemistries are frequently abnormal, with transaminases shown to be one-two times the upper limit of normal in most instances. The full spectrum of gastrointestinal involvement of the SARS-CoV-2 infection has yet to be fully seen.

Patient concerns: We present a case of a young woman with SLE who developed severe abdominal pain, nausea and vomiting, rapidly progressing to acute hepatic failure and tested positive for SARS-CoV-2 infection. She had no respiratory symptoms.

Diagnosis: A thorough work-up of acute liver failure including liver biopsy confirmed acute hepatitis with viral like changes. Common viral causes of liver failure were ruled out. The patient had no recent travel history.

Interventions: The patient was started on hydroxychloroquine due to SLE, treated with N-Acetyl-Cysteine, and methylprednisolone.

Outcomes: The patient improved with resolution of encephalopathy and normalization of her liver chemistries without any development of respiratory illness.

Conclusion: This case details a unique presentation of likely SARS-CoV-2 infection. Until now, the literature has primarily described a respiratory illness and liver injury with mild transaminase elevations. Significant liver injury progressing to acute liver failure should be considered in those with SARS-CoV-2 infection.

## 1 Introduction

Severe acute respiratory syndrome corona virus 2 (SARS-CoV-2) responsible for the COVID-19 pandemic has spread from Wuhan, China in December 2019 to 216 countries and territories as of September 10, 2020 with 27.74 million cases and 899,911 confirmed deaths.^[1]^ The spectrum of disease is most commonly seen as a viral pneumonia with high grade fevers, shortness of breath, dry cough, and chest pain with radiologic evidence of bilateral, interstitial, ground glass opacities and peripheral lung consolidation.^[2,3]^ Liver chemistries are frequently abnormal, with transaminases shown to be one-two times the upper limit of normal in most instances.^[4–6]^ The full spectrum of gastrointestinal involvement of the SARS-CoV-2 infection has yet to be fully seen. One case to date has been reported of a patient with SARS-CoV-2 infection, presenting first with acute hepatitis without respiratory symptoms.^[4]^ A second case of SARS-CoV-2 infection with development of severe liver failure has been published however development of hepatic failure ensued days into the disease course and introduction of antiviral therapy.^[5]^

We report a case of SARS-CoV-2 infection likely presenting as acute hepatitis rapidly progressing to fulminant hepatic failure without respiratory symptoms or evidence of drug induced liver injury. Written informed consent was obtained from the patient for publication of this case report and accompanying images.

## 2 Case

A 35-year-old woman presented to the emergency department with complaint of severe, diffuse abdominal pain. The pain started 1 day prior to arrival and progressed in severity with nausea, vomiting, and inability to tolerate fluids, prompting her to seek care in the emergency department. She denied cough, sore throat, shortness of breath, chest pain, myalgia, or anosmia. She lives with her husband and newborn child. She denied sick contacts and had not recently traveled. She had a past medical history significant for systemic lupus erythematosus for which she was taking prednisone 5 mg daily. She had previously been on hydroxychloroquine and mycophenolate mofetil which were being held while she was breast feeding. She had delivered a healthy newborn approximately 14 weeks prior to her presentation. Her outpatient medications included prednisone, ferrous sulfate, and etonogestrel implant. She denied the use of any over the counter medications including acetaminophen or non-steroidal anti-inflammatory medications. She did not use alcohol, recreational drugs, or tobacco products. Recent liver chemistries performed in the rheumatology clinic 2 months prior to presentation were normal.

On presentation, the patients temperature was 37.4^o^C, she was normotensive, tachycardic, and without hypoxia. She had scleral icterus, jaundice, and epigastric tenderness without evidence of encephalopathy. Her lung exam was unremarkable. Laboratory results on admission were as follows: Alkaline phosphatase 200 IU/L (N: <150), AST 219 IU/L (N: <35) ALT 278 IU/L (N: <55), total bilirubin 7.0 mg/dl (N: 0.2–1.2), direct bilirubin 5.5 mg/dl (N: <0.4), lipase 6 IU/L (N: <80), albumin 3.5 g/dl (N: 3.5–5.0), lactic acid 1.9 mmol/L (N: <2.2), white blood cell count 5.5 K/μl (N: 4.0–11.0) platelet count 55 K/μl (N: 140–400 K/μl), absolute lymphocyte count 0.7 K/μl (N: 0.8-4.1 K/μl), INR 1.5, ferritin 323 ng/dl (N: 5–200), CRP 66.8 mg/L (N: 0.0–8.0). SARS-CoV-2 rapid PCR by nasopharyngeal swab was collected given findings of lymphopenia, coagulopathy, and transaminase elevation which was positive for COVID-19 disease in the emergency department. She was masked and isolated per protocol. Viral causes for acute hepatitis were negative including hepatitis A, B, C, E, EBV, CMV, HSV 1, HSV 2, and HIV. Given the patients history of SLE, ANA was collected and positive. Mitochondrial antibody screen was weakly positive at 0.3 units (N: <0.1). Liver/kidney microsomal antibody and smooth muscle antibody serologies were normal. C3 and C4 were low and anti dsDNA was elevated to 392.2 IU from 58.3 IU 4 months earlier (N: 0.0–29.9). Abdominal ultrasound with Doppler revealed a normal appearing liver with stable hemangiomas and a patent portal and hepatic circulation. Severe gallbladder thickening with pericholecystic fluid without evidence of gallstones and a normal caliber common bile duct measuring 2.1 mm was also noted.

**Figure 1:**
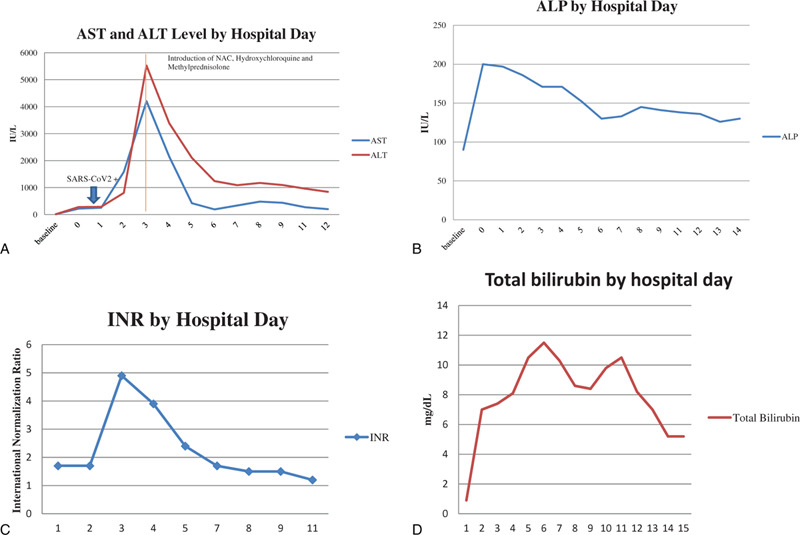
A: Transaminase Levels by Hospital Day- AST and ALT, Arrow indicating SARS-CoV2+ by PCR on Hospital day 0. Line indicates induction of NAC, hydroxychloroquine and methylprednisolone on hospital day 3. B: Transaminase Levels by Hospital Day- ALP. C: INR Levels by Hospital Day. D: Total Bilirubin levels in mg/dL by hospital day.

**Figure 2:**
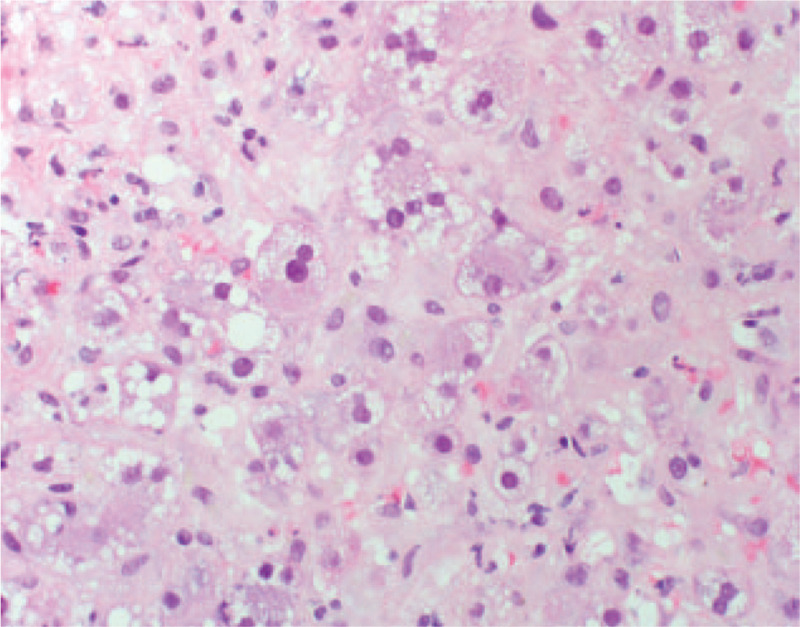
Transjugular liver biopsy showing giant cell transformation of hepatocytes.

**Figure 3:**
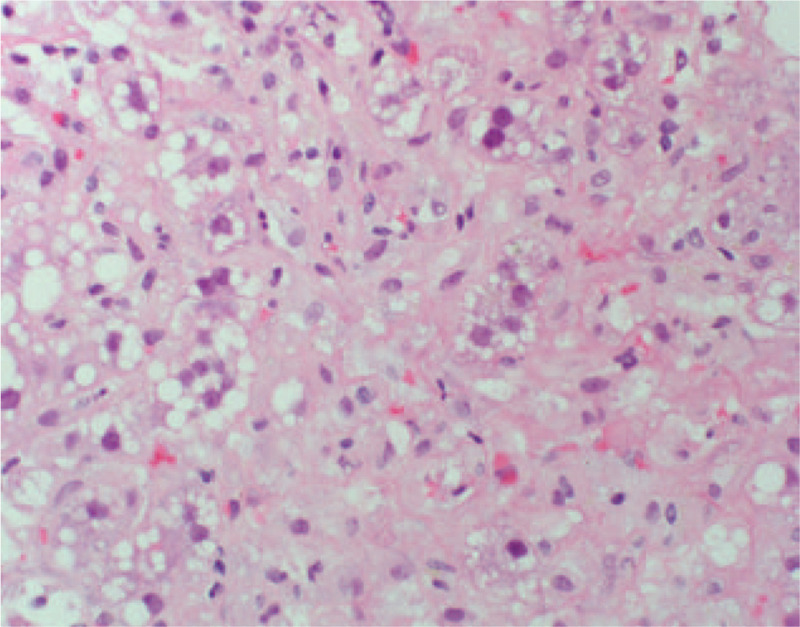
Transjugular liver biopsy showing multinucleated giant cells.

**Figure 4:**
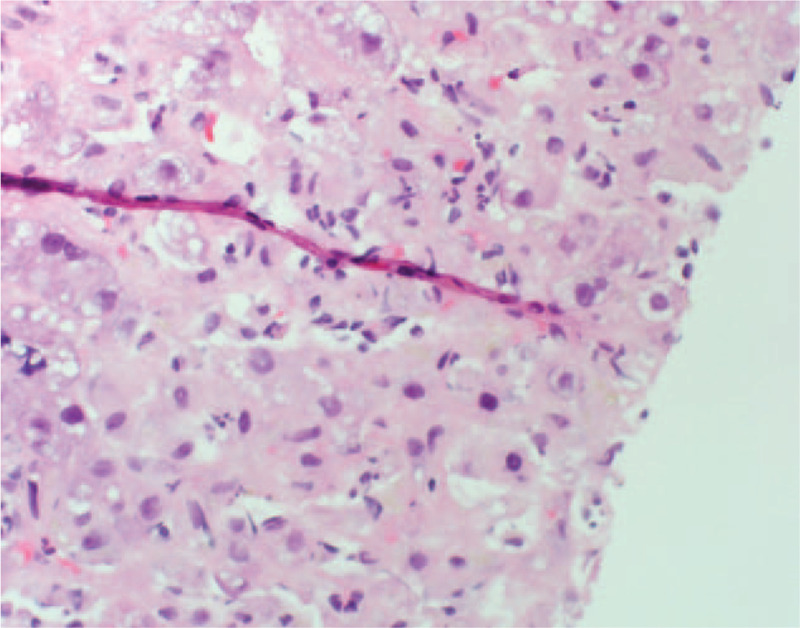
Transjugular liver biopsy showing acute lobular inflammation and focal necrosis.

**Figure 5:**
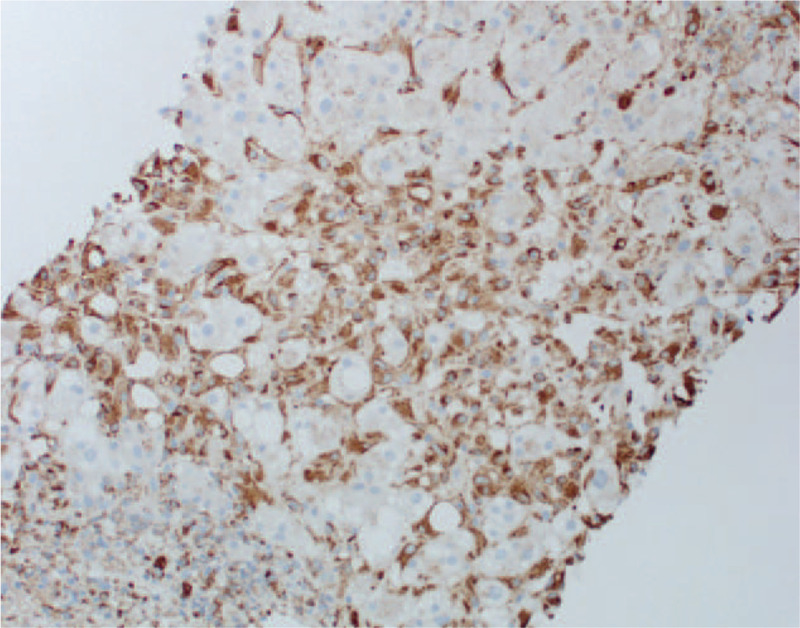
CD68 immunostaining of transjugular liver biopsy showing macrophages.

On hospital day 3, the patient developed mental status changes, anorexia, nausea, and vomiting with rising liver chemistries and coagulation studies (Fig. 1). These included alkaline phosphatase 171 U/L, AST 4202 U/L, ALT 5, 524 U/L, total bilirubin 10.5 mg/dl, INR 4.9. She had a mild cough, but otherwise normal respiratory exam. Chest x-ray on hospital day 3 was unremarkable. Hydroxychloroquine was initiated at 400 mg twice a day for 1 day, then continued at a dose of 200 mg twice a day to treat underlying SLE. Methylprednisolone was initiated at 40 mg IV daily on the chance the acute liver failure was induced by autoimmune hepatitis or a lupus associated hepatitis. As early treatment with N-acetyl cysteine has been shown to improve survival in those with non-acetaminophen liver failure, this was also initiated. Given the rapidly progressive and idiopathic nature of the acute liver failure, transjugular liver biopsy was performed which showed panacinar hepatitis with focal giant cell transformation, zone 3 necrosis, mild fatty change and focal hemophagocytosis (Figs. 2–5). These findings were most consistent with acute hepatitis and not consistent with autoimmune hepatitis, lupus associated hepatitis or biliary cholangitis. No viral inclusions were seen. SARS-CoV-2 tissue PCR or immunohistochemistry were not available at the facility at time of biopsy. On hospital day 4, the patient decompensated with grade IV encephalopathy and required intubation for airway protection. Over the next week, the patients liver chemistries and coagulation studies significantly improved (Fig. 1) and she was successfully extubated without difficulty with her mental status returning to baseline. Liver transplant was discussed however the patient was not felt to be a candidate due to the acute SARS-CoV-2 infection and significant improvement in mentation and coagulopathy after treatment with N-acetyl cysteine, methylprednisolone, induction dose of hydroxychloroquine, and supportive care.

## 3 Discussion

COVID-19 disease caused by SARS-CoV-2 has been defined as a respiratory illness presenting with dyspnea, fever, cough, anosmia, myalgias, and diarrhea. Severe infections leading to multisystem organ failure, systemic inflammatory conditions, stroke, cardiomyopathies, and rare cases of acute hepatitis have been reported. Several papers suggest evidence of liver injury or liver failure, however, acute liver failure is rare and typically occurs later in the course of COVID-19 disease, and may be influenced certain medications or high positive expiratory pressure as part of lung protective strategy during mechanical ventilation.^[1–4,8]^ The true spectrum of disease, however, is yet to be seen. Transaminase elevations have been noted in many individuals with SARS-CoV-2 infection, usually 1 to 2 times the upper limit of normal. In a cross sectional study of 417 patients who had developed transaminase elevation with SARS-CoV-2 infection, Qingzian et al demonstrated that most patients, >90%, presented with only mild elevations in transaminases, and only 24% progressed to transaminase elevations greater than 3 times the upper limit of normal (6). As our understanding of the breadth of illness in SARS-CoV-2 continues to emerge, we may see more cases with significant liver involvement including fulminant hepatic failure. To the best of our knowledge, this is the likely to be the first case report of acute liver failure associated with PCR confirmed SARS-CoV-2. This diagnosis requires the clinician to have a high level of clinical suspicion. Additionally, close monitoring of liver chemistries, synthetic functions, and mental status is imperative in those with SARS-COV-2. One case report of respiratory illness by SARS-CoV2 progressing to include acute liver failure has been reported in the setting of therapy with lopinavir/ritonavir. Our patient presented with severe hepatitis which rapidly progressed to acute liver failure prior to the onset of respiratory symptoms adding to the spectrum of illness that SARS-CoV-2 is able to cause. The patient did not experience hypoxemia or significant pneumonia, cardiac dysfunction nor have impairment in blood flow to or from the liver. With the exception of SARS-CoV-2, no other infectious etiologies to explain the acute liver failure were found.

The mechanism of liver injury by SARS CoV-2 is unknown. SARS-CoV-2 is a betacoronavirus^[2]^ which has shown affinity for binding to ACE2 receptors like the similar SARS-CoV and Middle Eastern Respiratory Syndrome responsible for the SARS pandemic^[2,7]^ and the MERS pandemic respectively.^[8]^ ACE2 receptors are known to be found on type 2 alveolar cells, up to 59.7% of cholangiocytes, and less commonly, hepatocytes (2.6%).^[6,7]^ It is reasonable to consider that SARS-CoV2 can bind to the ACE2 receptors of cholangiocytes inducing direct injury to bile ducts, acute liver injury, and even ALF. There is has been speculation that use of ACE inhibitors may cause upregulation of ACE2 receptors theoretically allowing worse injury by SARS-CoV2.^[5]^ This patient was not on ACE inhibitors.

Patients with SARS-CoV-2 are also experiencing severe inflammatory responses with marked cytokine storm which may induce liver inflammation and induce vascular thrombosis.^[6]^ Current literature supports the hypothesis that patients with higher transaminase levels on presentation, particularly in the hepatocellular pattern, have a higher likelihood of developing severe disease.^[6]^ This patient was diagnosed with a SLE flare during the hospital course by low C3, C4, and elevated dsDNA levels. The combination of these clinical features may have induced an overwhelming inflammatory response.

## 4 Conclusion

This case details a likely unique presentation of SARS-CoV-2 infection. Until now, the literature has primarily described a respiratory illness and liver injury with mild transaminase elevations. Significant liver injury, even progressing to acute liver failure should be considered in those infected with SARS-CoV-2.

## Author contributions

**Conceptualization:** Stephanie Melquist, Kayla Estepp, Yauheni Aleksandrovich, Angela Lee, Andrea Beiseker, John Bassett.

**Funding acquisition:** Stephanie Melquist.

**Resources:** Stephanie Melquist, Kayla Estepp, Yauheni Aleksandrovich, Angela Lee, Andrea Beiseker, Farid Saei Hamedani, John Bassett.

**Visualization:** Stephanie Melquist.

**Writing – original draft:** Stephanie Melquist, Kayla Estepp, Yauheni Aleksandrovich, Angela Lee, Andrea Beiseker, Farid Saei Hamedani, John Bassett.

**Writing – review & editing:** Stephanie Melquist, Kayla Estepp, Yauheni Aleksandrovich, Angela Lee, Andrea Beiseker, Farid Saei Hamedani, John Bassett.
